# Clinical Benefits and Limitations of Cone-Beam Computed Tomography in Endodontic Practice: A Contemporary Evidence-Based Review

**DOI:** 10.3390/diagnostics15243117

**Published:** 2025-12-08

**Authors:** Jasmine Wong, Chengfei Zhang, Angeline Hui Cheng Lee

**Affiliations:** Division of Restorative Dental Sciences, Faculty of Dentistry, The University of Hong Kong, Hong Kong SAR, China; jwong1@hku.hk (J.W.); zhangcf@hku.hk (C.Z.)

**Keywords:** CBCT, endodontics, clinical applications

## Abstract

Cone-beam computed tomography (CBCT) has transformed endodontic practice by enabling more precise diagnosis and treatment of pulpal and apical pathologies. The aim of this review was to summarize the current clinical applications, benefits and limitations of CBCT in endodontic practice. A search of electronic databases identified relevant literature on CBCT applications, innovations, and limitations. Emphasis was placed on identifying contemporary studies published in the last 5 years. In general, CBCT demonstrates better diagnostic efficacy across multiple applications, including identifying complex anatomy, detection of apical periodontitis, pre-surgical planning and the diagnosis and management of longitudinal root fractures, traumatic dental injuries and root resorptions. However, clinicians should balance the benefits of CBCT against its shortcomings, such as increased radiation exposure, presence of artifacts and higher costs. Proper use requires adherence to guidelines, optimized machine settings, and interpretation by trained individuals. Recent research explores the integration of CBCT with emerging technologies like artificial intelligence and guided systems. In summary, CBCT remains an essential tool for clinical decision-making in endodontics when used judiciously, with ongoing research continuing to expand its potential applications.

## 1. Introduction

Endodontics is an area of dentistry that is concerned with the diagnosis and management of pulpal and periapical diseases [1]. Accurate radiographic evaluation is one of the cornerstones of endodontics, and represents a crucial component of comprehensive endodontic diagnosis, treatment planning and outcome assessment [2]. Most of the radiographic criteria used in endodontics were originally developed using two-dimensional (2D) radiographic imaging, specifically, intra-oral periapical radiographs [3,4,5]. However, the shortcomings of 2D radiography include the overlapping of the surrounding anatomical structures, the tendency for dense cortical bone obscuring the underlying bone destruction, difficulties in identifying early mineral loss in bone and the inability to discern the buccal and lingual/labial positions of lesions [6,7,8]. These shortcomings have driven increased interest and enthusiasm for the applications of three-dimensional (3D) imaging.

Cone-beam computed tomography (CBCT) has emerged as a valuable imaging tool, gaining popularity in endodontics over the past two decades [9]. The generation of 3D images involves capturing and reconstructing multiple 2D images using a cone-shaped beam of radiation that rotates around the region of interest opposing a flat-panel detector. Unlike traditional CT scans, which use a fan-shaped beam to acquire a single slice per scan, CBCT captures the entire volume in a single rotation, enhancing efficiency and reducing patient radiation exposure [10].

CBCT offers numerous potential applications, given its ability to overcome many of the limitations of intra-oral radiographs [11]. However, CBCT also has drawbacks, such as increased radiation exposure, susceptibility to artifacts, and higher costs, which must be carefully weighed against its benefits [12]. As research evolves, our understanding of the potential clinical applications of CBCT imaging continues to expand. Therefore, this review aims to summarize the current evidence pertaining to clinical applications, benefits and limitations of CBCT in endodontics.

## 2. Methods

Relevant literature on CBCT applications, innovations and limitations was identified through electronic databases, i.e., PubMed, Scopus and Web of Science, using the following keywords and their combinations: ‘endodontics’, ‘CBCT’, ‘cone-beam computed tomography’, ‘diagnosis’, ‘outcome’, ‘apical periodontitis’, ‘surgical endodontics’, ‘root canal treatment’, ‘root canal retreatment’, ‘root fracture’, ‘resorption’ and ‘traumatic dental injuries’. The reference lists of identified reviews were also hand-searched. Emphasis was placed on identifying contemporary studies published after 2020. Nonetheless, landmark studies that made a significant contribution to the use of CBCT in endodontics were also recognized.

## 3. Radiological Aspects of CBCT in Endodontics

To establish an understanding of CBCT in endodontics, clinicians should appreciate basic radiological principles to effectively utilize and interpret CBCT images. The overall benefit gained from CBCT imaging depends largely on its diagnostic efficacy, which is particularly influenced by image resolution [13]. Voxel size is a primary factor that dictates resolution quality, with smaller voxels leading to better resolution. However, other acquisition parameters, such as higher tube voltage (kV) and tube current (mA), also play an important role [14,15]. With a wide variety of commercially available CBCT machines, voxel sizes can vary from approximately 0.075 to 0.4 mm^3^ [16]. Whilst smaller voxels can produce images of greater detail, this results in increased radiation dose, as machine parameters may automatically be adjusted to compensate for greater noise and artifacts associated with higher resolution [17]. This is supported by a meta-analysis indicating that high-resolution protocols invariably result in increased radiation dose [18]. A comparison of the effective doses from different sources is summarized in Table 1. These figures were based on a comprehensive summary of clinically measured effective doses reported by studies published after 2010 by Lee and colleagues (2021), as well as a publicly available report on radiation doses from different sources endorsed by the UK government [19,20]. The health and safety regulatory bodies of different countries may outline different protocols and radiation dose limits for the general population and professional personnel. Therefore, it is important to pay attention to the guidelines available, as well as obtain proper patient consent prior to exposing them to radiation.

Limited fields of view are often recommended for endodontic purposes [21,22,23]. Although what constitutes ‘limited’ can vary depending on the CBCT machine, a scan should ideally involve the tooth of interest and a few adjacent teeth [24]. This reduces the irradiated area, thereby reducing the effective dose [9]. When evaluating multiple teeth, the operator should carefully select an appropriate field of view, taking into account that scans with larger fields of view are associated with an increased effective dose—approximately 1.6 times higher—compared to scans with smaller fields of view [19,25]. In principle, CBCT images acquired for endodontic purposes should be taken with the lowest possible radiation dose that still yields acceptable image quality with sufficient diagnostic information [9,26], which would typically be a ‘small field of view’, approximately 40 mm^3^ to 50 mm^3^.

Artifacts are another limitation of CBCT image acquisition that can significantly compromise their diagnostic value. Discrepancies between the mathematical reconstruction of the data and the physical imaging process led to distortions of the patient’s anatomy in the CBCT image [27]. Commonly encountered artifacts include motion artifacts, noise, scatter and beam hardening or streaking [27,28]. A study investigating the quality of CBCT images used for endodontic purposes found that scatter artifacts were present in 100% of the samples, whilst beam hardening and motion artifacts were found in 94.7% and 7% of the images, respectively [28]. In particular, beam-related artifacts can cause significant image degradation and may be misinterpreted as root fractures or caries that are not actually present [29,30].

To prevent motion artifacts, patients should be encouraged to sit, rather than stand, with their eyes focused on a stationary image directly in front of them. Furthermore, for scans that require the disocclusion of teeth, patients may be instructed to occlude on a sturdy bite platform, rather than a soft cotton roll. These techniques aim to improve patient stability and image clarity [30,31]. During the scan, the patient should be observed for any obvious movements that would lead to significant motion artifacts, after which the clinician or technician can opt to terminate the scan prematurely as the image would likely be of insufficient diagnostic quality. Furthermore, metal artifact reduction algorithms may be employed by clinicians when the perceived risk of artifacts is high, such as in cases with metallic prosthetic work, orthodontic brackets and/or the presence of titanium implants. However, these algorithms are often prone to errors and may require 360° rotation scans, which can result in higher effective radiation doses [9,32,33]. Other methods to reduce such artifacts include removing or dismantling the metallic objects associated with the tooth of interest whenever possible [30,31].

Finally, adequate training and proficiency in CBCT interpretation are crucial for accurate assessment, as clinicians lacking such ability may result in improper diagnoses [34]. CBCT scans should ideally be evaluated on a high-resolution monitor display, with the entire scan systematically assessed in all planes. For the interpretation of a CBCT scan, guidelines have previously been published to promote a systematic and efficient workflow [35]. Key details to record include patient information, exposure parameters, and the anatomical region imaged. This evaluation should also include a summary of the teeth present, coronal and root canal status, presence or absence of any pathologies and peripheral findings [35]. However, when in doubt, the clinician should consider referral of the case to a radiologist for expert interpretation [9,21].

Currently, two position statements regarding the indications for CBCT imaging in endodontics have been published by the European Society of Endodontology (ESE) and the American Association of Endodontists (AAE). These guidelines serve as general recommendations for clinicians, endorsing the use of CBCT in diagnosis and pre-operative assessment, evaluation of complex anatomy, management of traumatic dental injuries, differentiation of various types of resorption, detection of vertical root fractures, identification of iatrogenic errors, and pre-surgical planning [9,21] (Table 2).

There is significant overlap of the suggested indications, albeit minor differences are noted as the AAE additionally touches upon the use of CBCT for implant planning and as a method for outcome evaluation. Ultimately, both guidelines emphasize a crucial element, that is, the benefits of the CBCT scan should outweigh the potential risks of radiation exposure. The ALARA principle, i.e., ‘as low as reasonably achievable’, must be considered when choosing the most appropriate machine parameters and situations for utilizing this imaging modality [26,36]. This approach will help ensure that the lowest possible effective dose is achieved while maximizing patient benefit across various clinical applications, which will be discussed in the following sections of this review.

## 4. Root Canal Morphology

The intricate morphology of the root canal system makes each root canal treatment unique. Features such as calcified canals, fins, isthmuses and other minute connections greatly influence the difficulty of the root canal treatment. Deciphering the root canal anatomy is a fundamental cornerstone to adequate chemo-mechanical debridement and ensuring favourable endodontic outcomes [37]. However, missed anatomy remains a common issue in endodontic therapy, accounting for up to 82.6% of endodontic failures associated with periapical pathosis annually [38,39]. Using the second mesio-buccal canal (MB2) of the upper molars as an example, periapical radiographs only revealed MB2 canals in 8% of teeth, whilst CBCT identified these canals in 62% [40]. It is the most commonly missed canal [41], yet the sole use of periapical radiographs often does not provide clinicians with sufficient information to locate them [42]. The high prevalence of these canals was further confirmed by a multicenter CBCT study and systematic review, which reported that MB2 occurs in 73.8% of maxillary first molars [43] and 39% of maxillary second molars [44].

CBCT has been recommended as a valuable tool for revealing these complex anatomical structures with great detail and precision [21,22]. Additionally, studies demonstrating a strong correlation between the root canal morphology observed in histological or sectioned samples and CBCT findings further confirm the imaging modality’s accuracy [45]. As CBCT is relatively non-invasive yet highly accurate, multiple large scale epidemiological studies have been conducted in the recent years to enhance our understanding of root canal morphology across populations from different demographic regions [43,44,46,47]. Smaller teeth, such as lower anterior incisors, may particularly benefit from CBCT assessment as a recent large-scale epidemiological study using CBCT imaging found the worldwide prevalence of lingual canals in mandibular incisors varied significantly between regions, ranging from 2.3% to around 50% [46]. Other complex anatomy often overlooked include mid-mesial canal in lower mandibular molars [48], disto-lingual root canal or radix entomolaris [49], C-shaped root canal morphologies [47], and developmental anomalies such as dens invaginatus [50].

Calcified canals pose another significant challenge for endodontists. CBCT has also been effectively utilized in the management of sclerotic anatomy and improved the clinician’s experience in treatment of such teeth [51]. The use of CBCT reduced stress levels in clinicians and resulted in more accurate assessment of canal morphology [51]. Furthermore, guidance of 3D imaging may lower the risk of overzealous dentine removal and iatrogenic errors, such as perforations, during treatment [52]. Excessive troughing can result in undesirable loss of tooth structure and increase the risk of root fracture, potentially compromising the tooth’s structural integrity [53]. Additionally, CBCT can be combined with virtual planning and guided systems to further guide clinicians in the detection of calcified canals [54]. Dynamic navigation facilitates real-time, intra-operative feedback through the 3D visualization of the canal morphology [55], minimizing guesswork and reduces the risk of iatrogenic errors, thereby enhancing both treatment effectiveness and efficiency [56]. Furthermore, in the age of minimally invasive dentistry, contracted access cavities that retire the concept of ‘straight line access’ have gained attention due to the potential to preserve peri-cervical dentine [57]. These access cavities demand immense precision and hence CBCT evaluation has been deemed an essential part of the pre-operative assessment [58]. Although there is still some debate on the long-term clinical implications of these types of accesses and whether they lead to improved fracture resistance [59], these knowledge gaps can guide the direction of future research.

In recent years, CBCT has been combined with deep-learning algorithms and artificial intelligence (AI), to streamline the pre-operative diagnosis of additional root canals and complicated anatomy [60,61]. Machine learning models have demonstrated high accuracy in using CBCT images to identify the MB2 canal, achieving an area under the curve (AUC) of nearly 90% precision [61]. Another AI algorithm showed impressive performance for detecting MB2 canals in obturated maxillary molars; however, authors cautioned the effects of artifacts and scattering in hindering diagnostic accuracy [60]. Recently, segmentation of the individual root canals from CBCT imaging was made possible via an AI-powered tool [62]. The evidence suggests that whilst CBCT has already been widely implemented as a method to identify complex root canal anatomy, AI models may play an enhancing role in this process in the future [60,61,62]. However, as with any emerging technology, clinicians should ensure that the programs have been subjected to external validation before implementing them in their clinical workflow, as well as comply with regulatory guidelines if available.

Clinicians should have sufficient knowledge of the root canal anatomy to fully reap the benefits of CBCT investigation [21,22]. Studies have shown that relying solely on CBCT to identify MB2 canals underestimates their presence by approximately 20% compared to when combined with selective clinical troughing, highlighting the importance of integrating imaging with clinical exploration for more accurate detection [40,63]. Additionally, its ability to reveal minute accessory canals or apical ramifications can be limited [64]. Micro-CT scans found that accessory canals have an average diameter of around 67 µm [64] which is likely too small for CBCT to detect reliably due to the voxel size limitations of many available machines [16,65]. Furthermore, the presence of radiopaque obturation materials and metallic restorations can reduce diagnostic accuracy due to scattering and metal artifacts [65]. Overall, guidelines stressed that CBCT should be used judiciously in appropriate cases rather than as a routine screening tool for root canal anatomy [21,22].

## 5. Diagnosis of Apical Periodontitis

The detection of apical pathology is a fundamental aspect of endodontic diagnosis and determination of treatment outcome [1]. Historically, most outcome studies and research on outcome predictors have relied on 2D imaging techniques [66,67]. However, in the recent years, there has been some debate as to whether 2D imaging provides sufficient information to properly ascertain treatment success. The visibility of apical lesions can be obscured by anatomical noise, such as the maxillary sinus, overlapping root apices, and thick buccal cortical plates [36,68]. Small lesions with minimal bone resorption are even more difficult to detect and this can induce disagreement between operators in the interpretation of these radiographs [69,70].

CBCT imaging overcomes many of the limitations associated with 2D radiography and reviews summarizing the current literature and recent outcome studies have suggested it can increase the detection of periapical radiolucencies by up to 40% [9,71,72]. A systematic review reported that the use of CBCT resulted in two-fold increase in odds ratio of detecting periapical lesions compared to periapical radiographs [12]. Multiple studies have also correlated CBCT findings with histological diagnoses, which serve as the gold standard [73,74]. Although both 2D and 3D imaging modalities were good at determining the absence of a lesion, periapical radiographs were associated with a higher rate of false negatives compared to CBCT [74]. Although parallax views with multiple radiographs could improve the diagnostic accuracy of periapical radiographs, CBCT demonstrates superior accuracy [73]. A recent review concluded that CBCT had a better agreement with histopathological findings than periapical radiographs [75]. A clinical study on cemental tears showed that CBCT also demonstrated a 40% higher detection rate of cemental tears associated with apical lesions compared to intraoral periapical radiographs [76].

Moreover, periapical lesions on conventional radiographs may appear smaller than on 3D imaging methods [36,77]. Despite an ex vivo study indicating that both periapical radiographs and CBCT had difficulty in detecting subtle mechanically prepared periapical lesions up to 0.8 mm in diameter, CBCT performed significantly better in identifying moderate (≥0.8–1.4 mm) and large (≥1.4 mm) lesions [8]. Another study assessed the accuracy of CBCT scans when measuring the dimensions of chemically prepared lesions and found CBCT yielded highly reproducible and accurate results with a deviation of less than 3% of the true diameter and depth of the lesions [78].

AI tools have further facilitated the diagnostic workflow of detecting apical periodontitis. Studies that have compared several AI models and manual segmentation by trained operators of CBCT scans with periapical lesions, it was found that the models were able to achieve up to 99% of diagnostic accuracy in identifying periapical lesions [79,80]. Additionally, AI has improved the diagnostic performance of dentists with varying levels of experience by not only reducing their assessment time but also increasing their clinical confidence [79]. AI-driven detection of various treatment parameters associated with endodontic treatment outcome, such as obturation quality and missed canals, has also shown promise as a valuable tool in clinical research [81].

Multiple outcome studies have used CBCT imaging as the assessment tool [72,82,83,84,85,86]. However, CBCT imaging has its own limitations. CBCT demonstrated reduced sensitivity in detecting mild bony changes associated with previously endodontically treated teeth compared to those in non-treated teeth due to artifacts [87]. A systematic review concluded periapical lesions associated with root-filled teeth are more susceptible to being obscured by artifacts, which negatively impacts the diagnostic performance of CBCT [75]. Furthermore, CBCT volumetric measurements of laboratory-created periapical lesions showed that minor measurement errors could accumulate, leading to discrepancies of up to 18% [88].

With the increased detection of periapical radiolucencies, concerns have been raised about the potential of over-diagnosis of apical periodontitis, which could lead to subsequent over-treatment of teeth. Clinically, healthy pulps may be associated with radiographic widening of the PDL space on CBCT, and researchers have debated where the threshold should be set when determining the radiographic signs of apical periodontitis [11,89]. Additionally, when the histological status of root apices that had previously undergone apical surgery was compared to CBCT findings, it was observed that 42% of teeth showing radiographic radiolucencies did not exhibit inflammation histologically but instead contained fibrous scar tissue [90]. This also has implications for how researchers and clinicians interpret endodontic outcome, as the increased detection of apical lesions may lead to the reporting of more treatment ‘failures’ when the true pathology is absent. This understanding allows researchers to scrutinize and re-evaluate the potential outcome predictors and treatment variables [71].

Some researchers have suggested the outcome evaluation via CBCT may require a different set of criteria, as lesions that persist after four years on CBCT could still be healing and should not necessarily be categorized as a failed or unfavourable outcome [71]. Ultimately, clinicians should exercise caution to avoid over-diagnosis and unnecessary retreatments by integrating radiographic findings with clinical assessments to establish the most appropriate diagnosis and treatment plan. Whilst periapical radiographs are still regarded as the default imaging modality in the diagnosis of apical periodontitis, CBCT is nonetheless a powerful tool, particularly in the presence of conflicting signs and symptoms [21,22]. Future research should aim to establish standardized criteria for defining “disease” in CBCT-diagnosed apical lesions and developing a consensus on the most accurate and reproducible methods for evaluating endodontic treatment outcomes in CBCT-based studies.

## 6. Surgical and Non-Surgical Retreatment

Despite primary non-surgical root canal treatment having a success rate of approximately 90% [91], some of the endodontically treated teeth may still experience treatment failure [67,92]. In such cases, root canal retreatment or apical surgery might be necessary to manage the endodontic diseases [93,94]. The additional information obtained from a CBCT scan has proven to be highly valuable for diagnosis and treatment planning. It enables clinicians to accurately assess the three-dimensional relationship of the tooth to surrounding vital structures, to determine the precise extent and location of any iatrogenic errors, if present, and to evaluate the level of retreatment difficulty.

For surgical endodontics, both ESE and AAE guidelines advocate the use of CBCT for pre-surgical planning. Appreciating the 3D relationship between the tooth and the surrounding anatomy facilitates optimal flap designs whilst avoiding unnecessary harm to surrounding vital structures, such as the maxillary sinus, mental nerve and inferior alveolar nerve [36,77,95]. Additionally, CBCT allows for detailed assessment of the buccal-lingual bone thickness and accurately depicts the extent of periapical lesions, which is particularly valuable in detecting cases with complete buccal dehiscences or root fenestrations and cortical bone involvement [96]. It also aids in the execution of the osteotomy and the option of planning advanced techniques such as bone windows [97]. Studies have also validated the accuracy of CBCT assessment in performing precise and reproducible anatomic measurements using CBCT-derived digital models [98].

Furthermore, in the era of digital dentistry and minimally invasive techniques, CBCT scans can be integrated with surgical stents to accurately guide the entry point of the surgical drill, enhancing precision in surgical procedures [99]. CBCT imaging coupled with computer-aided dynamic navigation can guide the angulation of root end preparations, leading to more conservative osteotomies, fewer iatrogenic errors and reduced operation time [100]. CBCT is also invaluable in autotransplantation planning by accurately assessing the root anatomy of the donor tooth and the suitability of the recipient site [101]. Moreover, guidelines have recommended fabricating a 3D-printed model of the donor tooth segmented from the CBCT data to minimize repeated try-ins of the donor tooth [64,102]. This approach helps prevent the periodontal ligament tissues from unnecessary damage, reduces the extra-oral dry time, and ultimately improves treatment outcomes [103,104].

For non-surgical retreatment, CBCT scans can help clinicians identify and evaluate various iatrogenic complications, including separated instruments and root perforations [21,22,105]. CBCT was found to have much higher sensitivity and specificity compared to periapical radiographs in the detection of perforations, particularly strip perforations, which are often obscured by the overlying intra-radicular and cortical bone [106]. For removal of separated instruments, the use of CBCT allows assessment of the location of the separated instrument, root canal curvature and proximity to vital structures [107,108]. This facilitates predictable instrument retrieval by allowing clinicians to select the most appropriate technique after evaluating the risks involved [108]. A study reported that more than half of clinicians who initially relied solely on periapical radiographs changed their approach to managing a fractured instrument after reviewing the CBCT image [107]. However, as previously mentioned, root filling materials such as gutta-percha and metallic posts can create streaking artifacts and beam hardening effects that impair diagnostic quality of the CBCT image [109].

Multiple studies have consistently found that clinicians tend to revise their treatment plans when new information from CBCT is available [110,111,112]. A study reported that there was an average difference of 62% in treatment plans when clinicians evaluated cases using only periapical radiographs compared to when they also had access to CBCT images [111]. Additional information provided by CBCT led to changes in at least half of the treatment decisions in other studies [110,112]. A systematic review concluded that CBCT information influences decision-making and prognosis assessment, especially in complex cases [113].

Despite these benefits, questions remain regarding whether the additional information from CBCT translates into improved endodontic outcomes [12]. According to Fryback and Thornbury (1991) [114], the value of diagnostic imaging tests is determined by several markers of efficacy, as demonstrated by their hierarchical model (Table 3) [115]. There are many studies showing the diagnostic value of CBCT and its ability to influence treatment plan, fulfilling level 1, i.e., technical efficacy [23,24], level 2, i.e., diagnostic accuracy efficacy [73,115], level 3, i.e., diagnostic thinking efficacy [111,112], and level 4, i.e., therapeutic efficacy [113]. However, there is a lack of evidence demonstrating that its use leads to improved outcomes, i.e., level 5, and societal benefit, i.e., level 6. Therefore, future research could focus on how the utilization of CBCT can enhance treatment outcomes and ultimately benefit society as a whole, thereby fully establishing the value of CBCT. In the meantime, clinicians should continue to exercise their professional and clinical judgment, utilizing CBCT selectively in cases where periapical radiographs are deemed insufficient for accurate diagnosis, proper treatment planning and effective management.

## 7. Vertical Root Fractures

Longitudinal root fractures, such as vertical root fractures, are difficult to diagnose accurately yet have a profound influence on the prognosis of a tooth and treatment plan [116]. For 2D radiography, a classic ex vivo study on extracted teeth with pre-existing fractures reported that fracture lines could only be depicted if the X-ray beam is within a margin of approximately 4 degrees [117]. Detection is further compromised if the degree of separation is minimal [118], coupled with the superimposition of anatomical structures that can obscure the fracture line. Diagnosis is made even more challenging due to the varying and inconsistent signs and symptoms associated with vertical root fractures [53]. Consequently, CBCT has been recommended to aid in diagnosis [21,22].

CBCT offers advantages over traditional radiographs by allowing clinicians to adjust viewing planes to better visualize potential fracture lines and overcoming issues of anatomical superimposition. Compared to periapical radiographs, an ex-vivo study on laboratory-created vertical root fractures of different diameters concluded that CBCT demonstrated higher accuracy in detecting vertical root fractures that are greater than 50 µm in diameter [118]. However, when fracture lines were narrower than 50 µm, both imaging modalities performed equally poor [118]. This is because when capturing the CBCT volume, voxel size plays an important role [119,120]. As some fracture lines may be as narrow as 10 µm [121], they may remain undetected due to the limits of the voxel parameters on the commercially available CBCT machines [119,121]. Other important parameters that can significantly influence the sensitivity of CBCT in the detection of vertical root fractures include exposure settings, speed and degree of rotation and image reconstruction variations [120].

Furthermore, vertical root fractures are typically associated with previously root canal treated teeth [122]. With the presence of root filling materials, beam hardening and scattering artifacts may easily be misinterpreted as fracture lines, whilst obscuring real signs of vertical root fractures [123]. An ex vivo study reported that even though CBCT were still more accurate than periapical radiographs, the presence of gutta-percha in the root canals significantly affected the diagnostic accuracy [121]. Despite recent advances in artifact reduction algorithms and motion correction techniques, a systematic review questioned their efficacy, noting that diagnostic accuracy in laboratory settings was actually better without activating such algorithms [124].

Overall, CBCT falls short when used to directly detect fracture line themselves, especially with root canal treated teeth. However, it is valuable for evaluating secondary changes associated with vertical root fractures, particularly when periapical radiographs provide inconclusive information [53]. These secondary changes often present in the bucco-lingual aspect and may include subtle crestal bone loss and widening of the periodontal ligament space [53]. That said, clinicians should exercise discretion when taking CBCT scans of previously root-filled teeth for detection of vertical root fractures, as the limitations of reconstruction and artifact presence may outweigh the benefits, and direct visualization through clinical examination remains a more reliable diagnostic approach [125].

## 8. Root Resorption

Root resorption is a pathological process that involves the progressive loss of the root cementum and dentine [126]. Early detection is challenging because the clinical and radiographic features vary depending on the type of resorption. Root resorption is classified into internal and external types. Internal resorption includes inflammatory and replacement forms, while external resorption encompasses inflammatory, surface, replacement, and cervical resorption [126].

As previously mentioned, periapical radiographs have their limitations for identifying early signs of root resorption. Extensive hard tissue deposits in the replacement forms of root resorption may obscure the outline of the resorptive defect, and identification of perforations, particularly in buccal and lingual aspects is almost impossible with a 2D planar image [41,127,128]. Moreover, differentiating between external and internal cervical resorption is particularly challenging due to similarity in 2D radiographic presentations [129]. In order to overcome these problems, parallax views through multiple periapical radiographs may be useful in determining the relative position of the resorptive defect, i.e., if the defect is located on the buccal or lingual root surface, and the relationship between the defect and root canal [129]. Despite so, periapical radiographs showed inferior diagnostic accuracy [115], leading to potential misdiagnosis and mistreatments.

CBCT has shown superior accuracy in diagnosing both internal and external root resorptions when compared to periapical radiographs [130]. Specifically, periapical radiographs achieved an area under curve value of 0.665, whereas CBCT attained a value of up to 0.990 [115]. It was also found that CBCT is superior at detecting resorptive lesions, even in root filed teeth [127]. Diagnostic clinical studies comparing CBCT and 2D radiographs have shown that about 30% of lesions are missed on periapical radiographs compared to CBCT imaging [131], and perforating lesions are more clearly visualized with CBCT [41]. In cases of multiple idiopathic resorptive lesions, full-mouth CBCT scans can provide a comprehensive assessment of the number and extent of the lesions [132]. To assess treatment outcome, CBCT has also been used as a tool for volumetric measurement of root resorptive defects in quantifying the extent of the lesion, aiding comparisons between the pre- and post-operative images [133].

CBCT has significantly influenced the diagnosis and management of external cervical resorption. The traditional classification by Heithersay (1996) [134] was based on 2D imaging, which often underestimated the true extent of lesions and the likelihood of unfavourable outcomes [134,135,136]. Incorporating CBCT data has led clinicians to re-evaluate their prognosis predictions in approximately one-third of cases, often downgrading cases from good to poor prognosis [136]. Clinicians also altered their treatment plan when provided with additional findings from CBCT scans, often resulting in a recommendation for extraction [135,137]. Patel and colleagues (2018) developed an updated classification system for external cervical resorptive lesions based on three observable parameters on CBCT scans: height, i.e., level along the root, circumferential spread and proximity to the root canal. This 3D classification allows reproducible and accurate assessment of the nature and extent of external cervical resorption, thereby guiding the treatment plan to optimize outcomes [138,139].

Several systematic reviews have concluded that CBCT is an important diagnostic tool in the evaluation and management of resorptive lesions [140,141]. Furthermore, both AAE and ESE recommendations echo the value of CBCT for the treatment of potentially restorable resorptive defects [21,22]. Except in cases where radiation exposure poses significant risks, such as in young patients, CBCT offers clear advantages over conventional periapical radiographs in assessing root resorption, making it an invaluable asset in modern endodontic diagnosis and treatment planning.

## 9. Traumatic Dental Injuries

Traumatic dental injuries require proper diagnosis and prompt management to optimize the chances of successful outcomes. Delays in treatment can lead to unfavourable consequences, such as pulp necrosis, root resorption, and ankylosis [142]. The current International Association of Dental Traumatology (IADT) guidelines recommend the use of CBCT for certain traumatic dental injuries when 2D radiographs do not provide sufficient diagnostic information. This is particularly relevant for injuries that may occur in multiple planes such as root fractures, crown-root fractures, and alveolar fractures [143].

Injuries such as horizontal root fractures tend to have an oblique orientation, making them less easily detected by parallel-oriented intra-oral radiographs [144]. Furthermore, if the separation of the fragments is narrow, the x-ray beam on a periapical radiograph may not traverse across the fracture line, potentially leading to missed detection [144]. An in-vitro study compared the detection of horizontal root fractures using CBCT scans versus digital and conventional periapical radiographs on extracted incisors with mechanically induced root fractures [145]. Unsurprisingly, the results concluded that the diagnostic accuracy and inter-observer agreement for conventional film radiographs was the lowest, whilst the diagnostic accuracy for CBCT image was the highest [145].

Intrusive and lateral luxation injuries tend to involve the buccal cortical alveolar bone fracture. The bucco-palatal position of the tooth and severity of fracture can only be fully appreciated using CBCT imaging [143]. This is supported by an in vivo study demonstrating that CBCT improved the diagnosis of various traumatic dental injuries, including lateral luxation, extrusion, and cortical plate fractures. The study found that CBCT images facilitated the detection of 99% of documented injuries, compared to only 84% with 2D imaging. Even when upper occlusal radiographs were taken in supplement to periapical radiographs, 2D modalities remained less diagnostically accurate [146].

The demographic that is most affected by traumatic dental injuries are children and young adolescents [147]. Given their increased sensitivity to radiation exposure, special precautions should be taken when employing CBCT as a diagnostic tool, such as low-dose protocols and ensuring limited fields of view [148]. Furthermore, clinicians who are not adept at utilizing and interpreting CBCT scans should refrain from prescribing them unnecessarily [22]. A study found that clinicians without proper training showed poor diagnostic performance when given CBCT images of traumatic dental injuries; in some cases, they even showed better diagnostic accuracy with 2D radiographs than 3D scans [34]. Nonetheless, with the correct application, CBCT can be used to accurately depict the extent, location and direction of these injuries and expedite the diagnostic process and provision of treatment [143].

## 10. Conclusions

In summary, the integration of CBCT into endodontic practice has significantly advanced the diagnosis, treatment planning, and management of complex dental conditions. Its capacity to produce multi-planar images addresses many of the shortcomings associated with traditional two-dimensional radiographs, enabling more precise detection and detailed assessment of complex anatomical structures and pathology. Nonetheless, the use of CBCT also involves considerations like higher radiation doses, susceptibility to artifacts, and increased costs, which require careful clinical judgment and adherence to current guidelines. Furthermore, judicious application of CBCT with critical appraisal of the available evidence is essential due to the inherent heterogeneity across CBCT machines and protocols, as well as publication bias. As ongoing research expands its applications and drives the development of adjunctive technologies, CBCT is increasingly becoming an invaluable tool in endodontics, significantly improving patient care and treatment decisions.

## Figures and Tables

**Table 1:** Average of effective radiation dose.

Type of Radiograph	Average Effective Dose (*μ*Sv)
Small CBCT [19] < 50 cm^2^	96.5 (17.1–220.2)
Medium CBCT [19] 50–150 cm^2^	113.7 (28.0 to 298.0)
Large CBCT [19] > 150 cm^2^	151.4 (32.0 to 392.2)
Intraoral [19]	1.32 (0.60–2.56)
Panoramic [19]	17.94 (3.47–75.0)
Transatlantic flight [20]	80
UK average annual radiation dose [20]	2700
USA average annual radiation dose [20]	6200

**Table 2:** Summary of the indications for CBCT according to recommendations by the European Society of Endodontics (ESE) and American Association of Endodontists (AAE)-American Academy of Oral and Maxillofacial Radiology (AAOMR).

	AAE-AAOMR [21] and ESE [22] Recommendation for the Indication of CBCT
Diagnosis and pre-operative assessment	To establish a definitive diagnosis in cases of nonspecific symptomatology where clinical findings and conventional two-dimensional radiography are inconclusive.
Anatomy	To visualize complex root canal systems, locate calcified or missed canals, and plan for guided endodontics.
Trauma	To identify and diagnose traumatic dental injuries that require advanced imaging modalities for proper assessment
Vertical root fractures	To aid in the diagnosis of vertical root fractures and the associated bone loss patterns when 2D radiography is inconclusive
Surgical	For preoperative assessment in periradicular surgery and to evaluate the proximity to adjacent anatomical structures.
Non-surgical retreatment	To investigate suspected etiologies of post-treatment disease and identify complications, such as perforations, separated instruments, and/or untreated anatomy.
Resorption	To localize, differentiate, and determine the prognosis of internal and external root resorption.
Post-operative assessment and outcome evaluation	To serve as a potential follow-up imaging modality if it was used for the initial evaluation.

**Table 3:** Fryback and Thornbury’s (1991) [114] hierarchical model for the efficacy of diagnostic imaging.

Level	Type of Efficacy
1	Technical efficacy
2	Diagnostic accuracy efficacy
3	Diagnostic thinking efficacy
4	Therapeutic efficacy
5	Patient outcome efficacy
6	Societal efficacy

## Data Availability

No new data were created or analyzed in this study. Data sharing is not applicable to this article.
